# Effects of Tail Vegetable Fermented Feed on the Growth and Rumen Microbiota of Lambs

**DOI:** 10.3390/ani14020303

**Published:** 2024-01-18

**Authors:** Rui Zhou, Lueyu Wang, Yaodong Li, Huihao Wu, Liping Lu, Rongxin Zang, Hongwei Xu

**Affiliations:** 1 College of Life Science and Engineering, Northwest Minzu University, Lanzhou 730100, China; zhour1222@163.com (R.Z.); 183061993@xbmu.edu.cn (L.W.); lyaodong@163.com (Y.L.); 901030@126.com (L.L.); 2 Experimental Teaching Department, Northwest Minzu University, Lanzhou 730100, China; wuhuihao99@163.com; 3 Key Laboratory of Biotechnology and Bioengineering of State Ethnic Affairs Commission, Biomedical Research Center, Northwest Minzu University, Lanzhou 730030, China

**Keywords:** fermented, intestinal microbiota, production performance

## Abstract

**Simple Summary:**

Numerous studies have emphasized the significance of providing starter feed to lambs in order to foster their growth and development. However, there has been a scarcity of research evaluating the capacity of fermented feed to supply lambs with the necessary nutrients for their development. This study aimed to assess the potential of fermented feed to regulate lamb development and influence their rumen microbial communities. Our findings indicate that incorporating fermented feed can enhance the average daily weight gain and final weight of lambs, facilitate rumen and small intestine development, optimize the rumen flora structure of lambs, and contribute to their overall growth and development. This lays a solid foundation for the healthy and rational feeding of lambs post-weaning and beyond.

**Abstract:**

This study explored the impact of integrating fermented feed into the starter diet of lambs, focusing on growth, health, serum antioxidants, immune markers, rumen fermentation, and microbial communities. Thirty-six ten-day-old female Tail Han lambs were randomly divided into three experimental groups, which were separately fed with alfalfa hay (LA group), tail vegetable fermented feed (LB group), and tail vegetable fermented feed supplemented with 0.1% microbial inoculants (LC group) during the experimental period. This study assessed the influence of fermented feed on various parameters, including growth performance, fiber degradation, rumen fermentation, enzymatic activities, and ruminal histomorphology. The results indicate that compared to the control group, the addition of fermented feed can increase the daily weight gain of lambs. Simultaneously, the addition of fermented feed can enhance the total antioxidant capacity of serum (*p* < 0.05). The addition of fermented feed promoted the increased height of villi in the duodenum or jejunum of lambs (*p* < 0.05), and the ratio of villi height to crypt depth in the LB and LC groups was also improved (*p* < 0.05). The addition of fermented feed increased the richness and diversity of the rumen microbial community in lambs (*p* < 0.05), primarily increasing the relative abundance of *Ruminococcus_1*, *Ruminococcaceae_UCG-005*, *Lachnospiraceae*, and *Lachnospiraceae_NK4A136_group*.

## 1. Introduction

The health and growth performance of lambs play a pivotal role in the economic viability of farming enterprises and the sustainable growth of livestock populations [1]. Research indicates that early dietary interventions and feeding strategies can significantly enhance lamb growth and health [2,3]. Starter feed, tailored to the digestive capabilities and enzymatic profile of young animals, supplies essential nutrients and trace elements crucial for their development, particularly around the weaning period [4,5].

Investigations have revealed that during the crucial weaning transition, pelleted starter feed is more effective in promoting gastrointestinal development, intestinal enzyme activities, nutrient absorption, and overall growth in lambs compared to traditional textured feeds. Current research is increasingly focusing on the nutritional composition and economic advantages of starter feed, along with its impact on physiological and biochemical markers and the gut microbiome in animals [6,7]. Studies by Casper et al. suggest a correlation between the energy content of starter feed and the development of the rumen in young ruminants; higher energy feeds lead to more pronounced rumen development [8]. Herawati et al. have underscored the significance of employing starter feed in the early stages to foster normal rumen development for optimal growth in lambs [9]. Given the limited variety of starter feeds in agricultural use, it is imperative to explore alternative approaches to maximize the benefits derived from starter feed ingredients.

Fermented feed, a sustainable and eco-friendly option, leverages the metabolic processes of microorganisms under controlled conditions. This process aims to reduce or transform anti-nutritional factors in animal and plant-based materials and minerals, resulting in a highly nutritious, non-toxic feed for livestock and poultry [3,10,11]. With the advancement of biotechnology, the use of bio-fermented feed has become increasingly prominent in animal nutrition research [12].

Therefore, in this investigation, we utilized a composite microbial agent, comprising three probiotics commonly employed in livestock farming, to ferment the starter feed. Our objective was to assess the impact of incorporating fermented feed into the starter diet on the production performance, meat quality, immune functions, and intestinal microbiota of Tail Han lambs.

## 2. Materials and Methods

### 2.1. Experimental Design and Animal Treatment

This study was approved by the Animal Welfare Committee of Northwest Minzu University, Lanzhou, China (XBMU-SM-2020010). Thirty-six ten-day-old female Tail Han lambs were randomly divided into three experimental groups, which were separately fed with alfalfa hay (LA group), tail vegetable fermented feed (LB group), and tail vegetable fermented feed supplemented with 0.1% microbial inoculants (LC group) during the experimental period (containing a balanced 1:1:1 ratio of live bacteria with a concentration of 4.7 × 10^8^ CFU/g). Each lamb is placed in a separate enclosure. During the experiment, the lambs were fed at regular intervals each day, and the amount of feed administered and the remaining feed for each lamb were recorded daily. After the feeding period, the lambs were allowed free access to maternal milk.

### 2.2. Preparation of Fermented Feed

*Bacillus subtilis* (CCTCC NO: 2021528), *Saccharomyces cerevisiae* (CICC NO: 1769), and *Lactobacillus plantarum* (CCTCC NO: M2021527) were sourced from the College of Life Science and Engineering at Northwest Minzu University. The preparation of the starter fermentation feed concentrate involved uniformly mixing these microbial agents in a 1:1:1 mass ratio. This mixture was then placed in a fermentation bag equipped with a breathing valve and left at room temperature for anaerobic fermentation over 5 days. The formulation of the starter feed adhered to the “Sheep Feeding Standard” (NY/T816-2004) [13]. The chemical composition of the feed for each treatment group of lambs is provided in Supplementary Table S1. The feeding experiment lasted for 50 days.

### 2.3. Growth Performance and Carcass Traits

Throughout the 50-day trial, lambs had unrestricted access to the experimental diet. Following an overnight fast, each lamb was weighed on an empty stomach at the beginning and end of the experiment. We meticulously recorded the average daily feed intake (ADFI) and average daily gain (ADG) and calculated the feed conversion ratio (FCR) as ADFI/ADG. Detailed observations and measurements were conducted for each lamb, including body length, height, chest circumference, depth, width, girth, and hip width.

### 2.4. Preparation and Sample Collection

On the 50th day, after weighing, lambs were anesthetized with sodium pentobarbital. Post-slaughter, we promptly extracted the rumen and small intestine, rinsing them with PBS buffer. We then measured the weight of each stomach compartment and recorded the length of each intestinal segment. The rumen, reticulum, omasum, and abomasum were separated. Rumen tissue samples, approximately 2 cm × 2 cm, were cut and immersed in 4% formaldehyde for histological analysis. Similar procedures were followed for the duodenum, jejunum, and ileum, with 10 cm sections sampled for histomorphological assessments. The Axio Lab. A1 microscope (Carl Zeiss, Jena, Germany) equipped with a Zeiss Axiocam ERc 5s digital camera was used for histological assessment. Images were analyzed and captured using ZEN 2.3 software (Carl Zeiss Microscopy GmbH, Oberkochen, Germany, 2011). Villus height (VH) and crypt depth (CD) were measured, and the V/C ratio (Villus height/Crypt depth, V/C) was calculated based on these measurements. The stomach coefficient was equal to stomach weight (g)/antemortem live weight (g) × 100%. The intestinal coefficient was equal to intestinal length (m)/antemortem live weight (kg). 

### 2.5. Serum Biochemical Indicators

We analyzed various serum parameters, including alanine aminotransferase (ALT), albumin (Alb), blood urea nitrogen (BUN), triglycerides (TG), aspartate aminotransferase (AST), total cholesterol (T-Chol), glucose (Glu), and total protein (TP). These were measured using the method detailed by Obeidat et al., involving centrifugation of serum samples at 2500× *g* at 4 °C [1].

### 2.6. Antioxidant Capacity

Blood samples were analyzed for antioxidant capacity. Commercial assay kits from Nanjing Jiancheng Bioengineering Institute were employed to assess levels of malondialdehyde (MDA) and the enzymatic activities of superoxide dismutase (SOD), catalase (CAT), glutathione peroxidase (GPX), as well as total antioxidant capacity (T-AOC).

### 2.7. DNA Extraction and High-Throughput Sequencing

For nucleic acid extraction, we employed the TGuide S96 Genomic DNA Extraction Kit from Tiangen Biotech Co., Ltd., Beijing, China. The process began with the use of diluted genomic DNA as a template for PCR amplification. We utilized KOD One TM PCR Master Mix and KOD FX Neo (TOYOBO) enzymes for high-fidelity and efficient amplification, specifically targeting the V3–V4 region of the 16S rDNA from rumen microbial DNA. The amplification employed barcode fusion primers: 338F (5′-ACTCCTACGGGAGGCAGCAG-3′) and 806R (5′-GGACTACHVGGGTWTCTAAT-3′). Subsequent steps involved repairing and end-repairing the amplified products, followed by adapter ligation using the SMRTbell template preparation kit from PacBio. The mixture was then purified using AMpure PB magnetic beads to recover the library for sequencing. The final library’s concentration was quantified using the Qubit detection system and its size with the Agilent 2100 system, ensuring a total amount exceeding the value calculated with SMRTlink. For sequencing, the library underwent primer and polymerase binding using the PacBio Binding kit, followed by purification with AMpure PB Beads. The samples were then sequenced on the Sequel (Pacbio, San Diego, CA, USA) platform. The raw sequence data underwent processing utilizing MOTHER. Subsequently, the clean reads were aggregated into operational taxonomic units (OTUs) employing a confidence threshold of 97%. The assessment of alpha diversity (specifically, OTU number and Shannon/Simpson’s diversity indices) and beta diversity (as determined via non-metric multidimensional scaling [NMDS]) was conducted using QIIME2. The structure of the rumen bacterial community was examined at the phylum and genus levels utilizing the Silva database (version 138) with a similarity cut-off of 70%. To pinpoint rumen bacteria unique to each dietary group, the linear discriminant analysis effect size (LefSe) tool was employed, with a linear discriminant analysis (LDA) score exceeding 4.0.

### 2.8. Statistical Analysis

All data were expressed as mean ± SEM and analyzed using the GraphPad Prism 8.0 software (San Diego, CA, USA). Comparative analyses employed one-way analysis of variance and multiple comparison tests for data from more than two groups. After correction, *p* < 0.05 was deemed statistically significant.

## 3. Results

### 3.1. Growth Performance

The impact of adding fermented feed to the starter feed on lamb growth performance is detailed in Table 1 and Table 2. Table 1 reveals a notable increase in body weight at 50 days for the LB group compared to the LA group, with a significant growth of 26.47% (*p* < 0.05). Furthermore, the LB group exhibited substantial improvements in average daily gain—29.21% and 44.35% higher than the LC group (*p* < 0.05). Table 2 shows that the LC group demonstrated significantly greater measurements in chest circumference, girth, rump width, chest width, chest depth, and girth index than the LA group (*p* < 0.05). Additionally, the chest circumference index in the LC group was notably higher than that in the LA group (*p* < 0.05). However, no significant differences were observed among the groups in terms of body height, body length, or body conformation index (*p* > 0.05).

### 3.2. Effects of Adding Fermented Feed in Starter on Serum Biochemical Indices of Lambs

The influence of adding fermented feed to starter feed on serum biochemical indices is summarized in Table 3. As per the data in Table 3, both ALT and ALP levels in the LB and LC groups were significantly lower than those in the LA group (*p* < 0.05). Conversely, levels of TP, ALB, BUN, GLU, TG, CHO, and CK showed no significant differences among the three groups (*p* > 0.05).

### 3.3. Antioxidant Capacity

Table 4 illustrates that the LC group experienced a substantial increase of 124.37% in serum GSH-Px content compared to the LA group. Additionally, the LB group showed a noteworthy increase of 64.56% in GSH-Px content relative to the LA group. The LC group also demonstrated a significant rise of 36.34% in GSH-Px content compared to the LB group. Regarding MDA levels, the LC group exhibited a reduction of 25.38% compared to the LA group, while the LB group showed a decrease of 35.76% relative to the LA group. 

### 3.4. Influence on Gastrointestinal Tract Development

The impact of adding fermented feed to the starter on gastrointestinal tract development is presented in Table 5, Table 6 and Table 7. Table 5 reveals no significant differences in rumen coefficients among the experimental groups and the LA group (*p* > 0.05). However, there was an upward trend in rumen coefficients in the LA, LB, and LC groups, with respective increases of 12.71% and 33.52%. The reticulum, omasum, and abomasum coefficients showed no significant variation among the groups (*p* > 0.05). The LC group exhibited a significant 73.68% increase in the duodenal coefficient compared to the LA group (*p* < 0.05). The jejunal coefficient in the LB group increased by 48.66% in comparison to the LA group (*p* < 0.05), and the LC group registered a 30.30% rise compared to the LA group (*p* < 0.05). The ileal coefficient in the LB group was significantly higher by 220.00% than that in the LA group.

The LC group’s duodenal villus height significantly surpassed that of the LB and LA groups (*p* < 0.05), with increases of 21.89% and 70.73%, respectively. The LB group displayed a 40.06% increase over the LA group. In terms of duodenal crypt depth, both the LC and LB groups showed significant reductions compared to the LA group, by 30.47% and 25.80% (*p* < 0.05), respectively. There were marked differences in the villus height to crypt depth (V/C) ratio among the three groups in the duodenum (*p* < 0.05). The LC group’s V/C ratio was 148.71% higher than the LA group, and the LB group was 89.74% higher than the LA group. Additionally, the LC group exhibited a 31.08% increase over the LB group.

Significant differences in jejunal villus height were observed among the three groups (*p* < 0.05). The LC group registered a substantial increase of 63.74% compared to the LA group, while the LB group experienced a notable increase of 35.86% relative to the LA group. Additionally, the LC group’s jejunal villus height was 20.51% greater than that of the LB group. The crypt depth in the LC group was considerably lower than in the LA group, showing a 22.35% decrease (*p* < 0.05). In terms of the V/C ratio in the jejunum, notable differences were evident among the groups (*p* < 0.05), with the LC group displaying an increase of 110.37% over the LA group and the LB group showing a 52.59% increase compared to the LA group. The LC group also exhibited a 37.86% higher V/C ratio than the LB group. In the ileum, the villus height in the LC and LB groups was significantly elevated, by 36.47% and 28.38%, respectively, compared to the LA group (*p* < 0.05).

### 3.5. Microbial Composition in the Rumen

**Figure 1:**
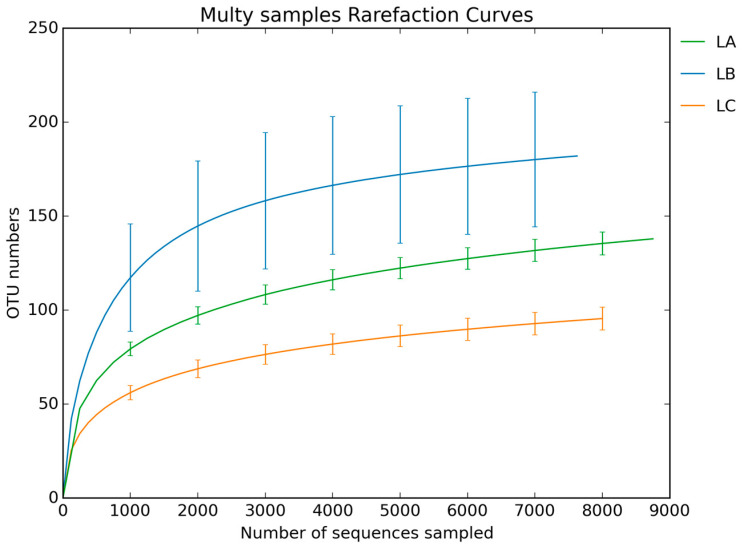
Dilution curve for 16S rDNA gene sequencing.

**Figure 2:**
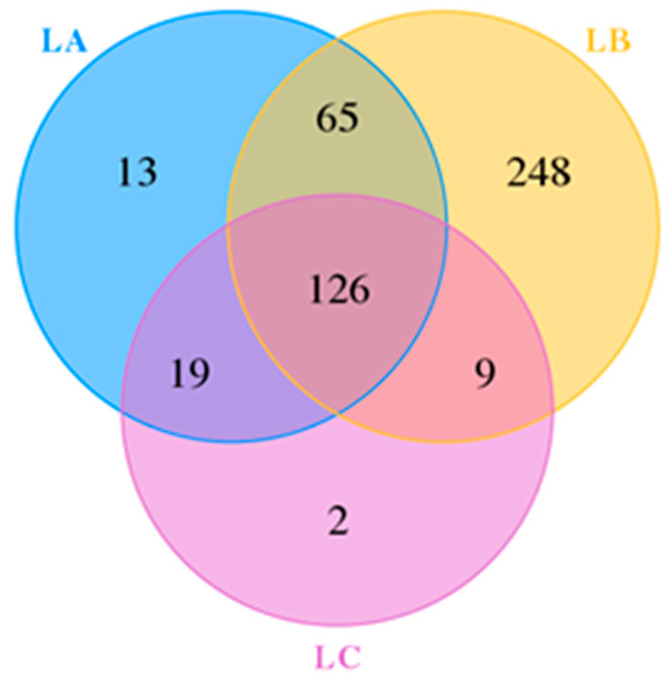
The Venn analysis of OTU.

The dilution curve (Figure 1) illustrates a plateau phase, indicating that the sequencing data are comprehensive and near saturation. The analysis in Figure 2 revealed a total of 482 OTUs across the groups, with 126 OTUs common to all, accounting for 26.14% of the total. The LC group had 13 unique OTUs, the LB group 248, and the LA group 2, suggesting greater species richness and bacterial diversity in the experimental groups compared to the LA group.

**Figure 3:**
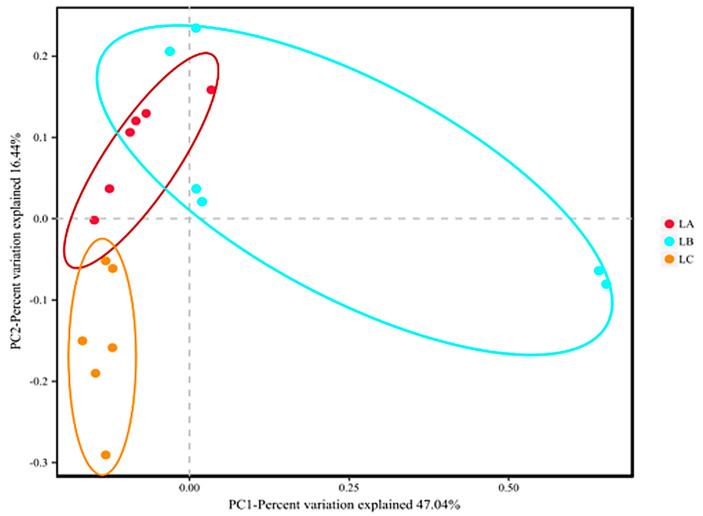
Principal coordinate analysis (PCoA) of microbial structure from the cecal digesta between LA, LB, and LC groups (based on the BrayeCurtis distance).

Principal coordinate analysis (PCoA), as shown in Figure 3, revealed distinct beta diversity between the sample groups, with the LA group markedly separate from the LB and LC groups. This separation indicates some overlap in species’ composition between the LA group and the experimental groups, yet with significant differences between species (*p* < 0.05).

**Figure 4:**
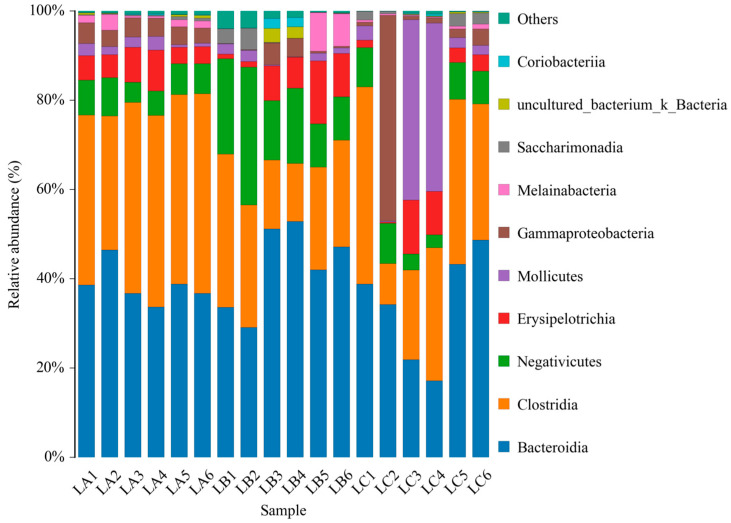
The abundance of intestinal microbiota at phylum levels of sheep.

As per Figure 4, at the phylum level, the LC group exhibited a significantly higher abundance of *Firmicutes* (31.54%) compared to the LA group (*p* < 0.05). No significant differences were noted between the LB group and the other groups in terms of *Firmicutes* abundance (*p* > 0.05). The abundance of Bacteroidetes in the LB and LC groups was lower than in the LA group, though not significantly (*p* > 0.05). *Tenericutes* were most abundant in the LA group, while Spirochaetes were predominantly found in the LB group.

**Figure 5:**
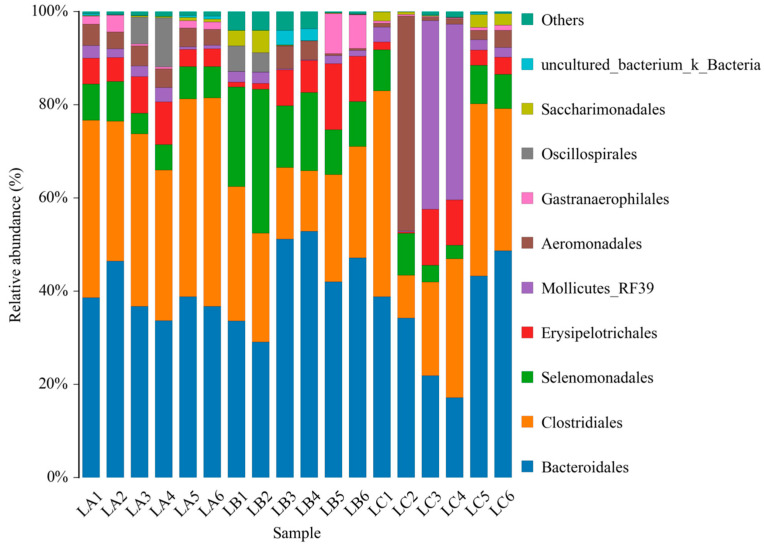
The abundance of intestinal microbiota at genus levels of sheep.

Figure 5 details the microbial composition at the genus level across the three groups. *Prevotella-7* emerged as the dominant genus, with respective abundances of 20.35%, 12.34%, and 32.49% in the LC, LB, and LA groups, respectively. The prevalence of *Prevotella-7* in the LC group was significantly higher than in the LB group (*p* < 0.05), but similar to the LA group. The LA group exhibited a notably higher abundance of *Prevotella* compared to the LC group (*p* < 0.05). *Prevotella-1* was significantly more abundant in the LB group than in both the LA and LC groups (*p* < 0.05).

## 4. Discussion

The starter feed plays a crucial role in supplying trace elements and essential nutrients necessary for the growth and development of juvenile animals, especially during the early weaning stage [2,14,15]. Research indicates that the early introduction of starter feed can promote rumen development and facilitate early weaning [16]. However, the variety of starter feeds available is limited, posing a challenge to enhancing their nutritional composition and promoting juvenile animal development [17]. Fermentation, a natural process, has been recognized for its ability to increase the nutritional value of feed by enhancing protein, vitamins, essential amino acids, and antioxidant capacity while also improving flavor [18,19,20]. Bio-fermented feed, rich in small molecular substances, various unsaturated fatty acids, and aromatic compounds is readily absorbed by the animal’s intestinal tract, enhancing palatability and increasing feed intake [21]. This experiment aimed to investigate the impact of a fermented starter diet of tail vegetables on the growth performance, serum parameters, antioxidant capacity, and rumen microflora of lambs.

The experiment revealed that the final weight of the LC group was significantly higher than that of the LA group. Additionally, both the LB and LC groups demonstrated a higher average daily gain when contrasted with the LA group. These findings are consistent with prior research, indicating that integrating fermented feed into the initial diet contributes to an enhanced growth performance in lambs [22,23]. The enhancement is likely attributed to the beneficial bacteria present in the fermented feed, which promote digestive tract development in lambs and aid in breaking down macromolecular feed components into smaller, more absorbable molecules, consequently improving feed utilization efficiency.

In this experiment, the indexes of chest circumference, abdominal circumference, hip width, chest width, chest depth, and abdominal circumference in the LC group were significantly higher than those in other groups. However, there were no significant differences in height, body length, body mass index, and body length index among the three groups, and all the body measurements were within the normal range. The results showed that the fermented feed of tail vegetable could effectively improve the growth performance of lambs. This effect may be attributed to the fact that probiotics enable lambs to effectively absorb nutrients from fermented feed, thus improving feed utilization.

Blood metabolites, produced during metabolism, serve as crucial indicators of animal health, nutrient utilization, and overall metabolic activity [24,25]. Serum triglycerides (TG) and cholesterol (CHO) levels are key to assessing lipid metabolism, with higher concentrations indicating increased fat deposition [26,27]. In this study, the LC group exhibited a 14.81% reduction in serum TG content compared to the LB and LA groups and a 12.90% decrease compared to the LA group alone. These findings suggest that fermented feed positively regulates lipid metabolism, with fermentation breaking down crude fat into smaller molecules for better absorption. Total protein (TP) in serum reflects an animal’s protein metabolic capacity, while albumin (ALB), synthesized by liver parenchymal cells, maintains plasma osmotic pressure [28,29]. This study found no significant differences in TP and ALB levels across the three groups, all within acceptable ranges. This indicates that fermented feed does not significantly impact the immune system of lambs. Blood enzymes in livestock primarily originate from various tissues and organs, with alanine aminotransferase (ALT) and alkaline phosphatase (ALP) being critical transaminases representing liver health [30,31]. Elevated levels of these enzymes in the bloodstream often signal liver damage. In our experiment, the LC and LB groups showed significantly lower ALT levels than the LA group, 52.51% and 58.73%, respectively, with no notable difference between the LC and LB groups. Additionally, the LC group’s serum ALP level was reduced by 57.15% compared to the LA group, while the LB group saw a 38.08% decrease compared to both the LA and LB groups. These results indicate that fermented feed offers effective liver protection and plays a significant role in liver development.

Antioxidant enzymes maintain a balance between oxidation and antioxidation when animals are in a normal physiological state. However, when the body is stimulated by various stimuli, oxidative stress will occur, which will cause damage to tissues and cells, resulting in the decrease of T-AOC, T-SOD, and GSH-Px [26,27]. Excessive free radicals will disrupt the oxidative balance in the body, leading to excessive oxidation, and eventually leading to apoptosis [1,4]. The results showed that the content of GSH-Px in the serum of the LB group and LC group was significantly higher than that of the LA group. The results indicated that the feeding of a fermented diet could improve the antioxidant capacity of lambs. In addition, the use of microbial agents promotes the absorption of fermented feed by lambs, further improving the antioxidant capacity of lambs, thus stabilizing the physiological state of the body and promoting the growth performance of lambs.

The rumen microbial community is instrumental in maintaining lamb health through nutrient metabolism, mucosal barrier integrity, and defense against pathogenic invasions [32,33]. In our study, we sequenced and analyzed rumen digesta to assess the gut microbiota’s species richness. Comparative analysis of various diversity indices, including Shannon, ACE, Sobs, and Chao, revealed that fermented feed enhances the relative abundance of rumen microbes in lambs. Moreover, our beta diversity analysis indicated distinct intestinal microbial communities between the LA, LB, and LC groups. This variation is likely attributable to the antimicrobial properties of probiotics in the fermented feed. *Firmicutes*, a phylum of critical bacterial, play a vital role in the production of short-chain fatty acids (SCFAs), providing an efficient energy source for intestinal cells and augmenting carbohydrate absorption in the host [34]. In this study, the *Firmicutes* to *Bacteroidetes* ratio (F:B) was dose-dependently reduced by the inclusion of fermented feed. Such a diet increased the relative abundance of *Firmicutes* and decreased the prevalence of *Bacteroidetes*, favoring the digestion of polysaccharide-rich feed and mitigating intestinal inflammatory diseases [35]. The genus *Ruminococcus*, found in the rumen, produces significant quantities of cellulases, hemicellulases, and xylanases, which are crucial for degrading cellulose and hemicellulose in coarse feed. Our findings suggest that the supplementation of fermented feed elevates the relative abundance of *Ruminococcus* in the rumen, and is potentially linked to enhanced productivity in lambs [36].

## 5. Conclusions

This study ultimately demonstrated that the addition of fermented feed in the starter diet significantly improved the growth performance of lambs. The primary benefits of this feed included an increase in the average daily weight gain and final body weight of the lambs. Furthermore, this study validated that this dietary approach enhanced the lambs’ antioxidant capacity and improved the tissue morphology of their rumen and small intestines, thereby promoting more efficient digestion and absorption processes. Additionally, the addition of fermented feed in the starter diet had a positive impact on the rumen microbial community. This feed optimized the species richness and diversity of microbes in the lambs’ guts, increasing the proportion of beneficial microbes while reducing the presence of potentially harmful species. These changes in the rumen microbial structure played a crucial role in promoting comprehensive growth and development in lambs.

## Figures and Tables

**Table 1:** Effects of tail vegetable fermented feed on growth performance of lambs.

Item	Treatments			SEM	*p*-Value
	LA	LB	LC		
Initial weight (kg)	5.86 ± 0.44	5.76 ± 0.31	5.76 ± 0.54	0.24	0.546
Final weight (kg)	15.26 ± 0.78 ^b^	17.90 ± 1.11 ^ab^	19.30 ± 1.15 ^a^	1.70	0.043
Average daily gain, g/d	187.59 ± 6.55 ^b^	242.40 ± 4.58 ^a^	270.80 ± 7.89 ^a^	15.7	0.013
Average daily feed intake, g/d	1715.9 ± 26.44 ^b^	1874.5 ± 16.78 ^a^	1857.9 ± 16.28 ^a^	2.52	0.048
Feed conversion rate	9.17 ± 0.14 ^b^	7.74 ± 0.07 ^a^	6.94 ± 0.05 ^a^	0.43	0.035

SEM, standard error of the mean. ^a,b^ Different superscript letters in each indicator represent a significant difference (*p*-value < 0.05).

**Table 2:** Effects of tail vegetable fermented feed on lamb body size.

Item	Treatments			SEM	*p*-Value
	LA	LB	LC		
Body height/cm	55.00 ± 1.75	58.50 ± 1.60	58.00 ± 1.52	0.756	0.042
Body length/cm	53.66 ± 1.58	54.33 ± 2.33	58.00 ± 0.57	0.565	0.026
The chest/cm	57.00 ± 1.15 ^b^	56.16 ± 1.01 ^b^	64.00 ± 2.64 ^a^	0.781	0.046
Pipe surrounds/cm	5.83 ± 0.16 ^b^	6.00 ± 0.00 ^b^	7.00 ± 0.34 ^a^	2.563	0.043
Tajiri wide/cm	12.16 ± 1.20 ^b^	15.33 ± 0.60 ^b^	17.33 ± 0.60 ^a^	0.876	0.017
Chest width/cm	12.16 ± 0.44 ^b^	12.16 ± 0.72 ^b^	20.66 ± 0.66 ^a^	4.523	0.023
Chest deep/cm	23.83 ± 0.83 ^b^	25.16 ± 1.01 ^b^	29.66 ± 1.76 ^a^	2.869	0.012
Trunk indices/%	106.26 ± 1.72	103.80 ± 5.27	110.39 ± 4.98	2.538	0.406
Length indices/%	97.62 ± 3.30	93.17 ± 5.59	100.12 ± 2.56	1.957	0.634
Chest indices/%	96.19 ± 3.78 ^b^	103.73 ± 1.98 ^ab^	110.29 ± 2.52 ^a^	3.456	0.023
Tube circumference indices/%	10.60 ± 0.04 ^b^	10.26 ± 0.27 ^b^	12.05 ± 0.31 ^a^	1.563	0.015

SEM, standard error of the mean. ^a,b^ Different superscript letters in each indicator represent a significant difference (*p*-value < 0.05).

**Table 3:** Effects of tail vegetable fermented feed on serum biochemical indexes of lambs.

Item	Treatments			SEM	*p*-Value
	LA	LB	LC		
TP (g/L)	66.83 ± 1.95	67.40 ± 1.68	72.33 ± 1.92	3.153	0.073
ALB (g/L)	30.50 ± 1.21	31.73 ± 1.61	34.20 ± 1.00	0.456	0.085
ALT (U/L)	26.66 ± 1.33 ^a^	12.66 ± 0.33 ^b^	11.00 ± 1.15 ^b^	3.415	0.001
ALP (U/L)	669.00 ± 17.47 ^a^	463.00 ± 17.03 ^b^	286.66 ± 14.03 ^c^	25.15	0.023
BUN (mmol/L)	6.14 ± 0.56	5.68 ± 0.25	4.04 ± 0.37	0.165	0.151
GLU (mmol/L)^-^	5.14 ± 0.21	4.95 ± 0.72	5.06 ± 0.39	1.016	0.084
TG (mmol/L)	0.31 ± 0.04	0.27 ± 0.05	0.23 ± 0.02	0.125	0.081
CHO (mmol/L)	1.30 ± 0.14	1.16 ± 0.07	1.22 ± 0.10	0.054	0.076
CK (U/L)	142.33 ± 3.71	138.00 ± 9.29	137.33 ± 3.52	0.578	0.085

SEM, standard error of the mean. ^a,b,c^ Different superscript letters in each indicator represent a significant difference (*p*-value < 0.05).

**Table 4:** Effects of tail vegetable fermented feed on antioxidant capacity of lambs.

Item	Treatments			SEM	*p*-Value
	LA	LB	LC		
Total antioxidant capacity U/mL	2.59 ± 0.18 ^c^	2.83 ± 0.07 ^b^	3.32 ± 0.49 ^a^	0.456	0.042
Total superoxide dismutase, U/mL	181.12 ± 6.73	196.76 ± 8.75	191.34 ± 4.25	3.565	0.066
Glutathione peroxidase, U/mL	87.75 ± 3.52 ^c^	144.41 ± 6.70 ^b^	196.89 ± 3.03 ^a^	12.78	0.036
Malondialdehyde, nmol/mL	2.60 ± 0.16	1.67 ± 0.18	1.94 ± 0.43	0.576	0.075

SEM, standard error of the mean. ^a,b,c^ Different superscript letters in each indicator represent a significant difference (*p*-value < 0.05).

**Table 5:** Effects of tail vegetable fermented feed on stomach recovery coefficient of lambs.

Item	Treatments			SEM	*p*-Value
	LA	LB	LC		
Rumen coefficient	1.73 ± 0.26	1.95 ± 0.10	2.31 ± 0.24	0.456	0.062
Reticulum coefficient	0.26 ± 0.05	0.31 ± 0.04	0.26 ± 0.05	0.565	0.086
Flap coefficient	0.18 ± 0.03	0.21 ± 0.02	0.23 ± 0.01	2.563	0.076
Abomasum coefficient	0.57 ± 0.14	0.68 ± 0.06	0.70 ± 0.03	0.876	0.075

SEM, standard error of the mean.

**Table 6:** Effects of tail vegetable fermented feed on rumen morphology of lambs.

Item	Treatments			SEM	*p*-Value
	LA	LB	LC		
Height of rumen papilla	1402.46 ± 50.90 ^c^	1887.23 ± 15.91 ^b^	2989.76 ± 115.49 ^a^	0.456	0.042
Ruminal papilla width	545.46 ± 18.70 ^b^	666.90 ± 67.77 ^a^	667.76 ± 31.23 ^a^	0.565	0.026
Thickness of the rumen muscle layer	921.33 ± 22.11 ^b^	878.90 ± 23.78 ^b^	1028.40 ± 11.16 ^a^	2.563	0.046
Rumen thickness	1086.16 ± 39.61 ^b^	1263.26 ± 61.34 ^ab^	1429.13 ± 62.94 ^a^	0.876	0.035

SEM, standard error of the mean. ^a,b,c^ Different superscript letters in each indicator represent a significant difference (*p*-value < 0.05).

**Table 7:** Effects of tail vegetable fermented feed on the morphology of lambs’ small intestines.

Item	Treatments			SEM	*p*-Value
	LA	LB	LC		
Villus height (V)					
Duodenum, mm	397.63 ± 9.04 ^c^	556.96 ± 13.70 ^b^	678.90 ± 19.33 ^a^	2.045	0.016
Jejunum, mm	449.83 ± 10.00 ^c^	611.16 ± 3.18 ^b^	736.56 ± 7.70 ^a^	3.078	0.001
Ileum, mm	427.66 ± 16.34 b ^b^	549.03 ± 20.95 ^a^	583.63 ± 7.92 ^a^	5.468	0.001
Crypt depth (C)					
Duodenum, mm	337.80 ± 4.51 ^b^	250.63 ± 12.51 ^b^	234.86 ± 16.87 ^b^	0.814	0.032
Jejunum, mm	333.50 ± 10.66 ^a^	300.06 ± 27.28 ^ab^	258.96 ± 5.60 ^b^	0.054	0.001
Ileum, mm	276.96 ± 15.02	264.83 ± 26.24	298.80 ± 43.02	0.785	0.081
V:C ratio					
Duodenum, mm/mm	1.17 ± 0.02 ^c^	2.22 ± 0.10 ^b^	2.91 ± 0.23 ^a^	0.695	0.04
Jejunum, mm	1.35 ± 0.06 ^c^	2.06 ± 0.17 ^b^	2.84 ± 0.05 ^a^	0.478	0.03
Ileum, mm	1.55 ± 0.11	2.10 ± 0.16	2.04 ± 0.32	0.896	0.072

^a,b,c^ Different superscript letters in each indicator represent a significant difference (*p*-value < 0.05). SEM, standard error of the mean.

## Data Availability

Raw data for the figures are available upon reasonable request from the corresponding author.

## Supplementary Materials

The following supporting information can be downloaded at: https://www.mdpi.com/article/10.3390/ani14020303/s1, Table S1: Experimental diet composition and nutrition level (air-dried basis).
