# Supplementary material for: Effects of Tail Vegetable Fermented Feed on the Growth and Rumen Microbiota of Lambs
Source: Animals (Basel). 2024 Jan 18;14(2):303. doi: 10.3390/ani14020303 (PMC10812633; doi:10.3390/ani14020303)
Supplement: Supplementary file 1 [file animals-14-00303-s001.zip › animals-2778878-supplementary.pdf]

**Table S1.** Experimental diet composition and nutrition level (air-dried basis).

| **Items** | **LA** | **LB** | **LC** |
|---|---|---|---|
| Ingredients | | | |
| Corn straw (%) | 33.70 | 36.6 | 43.40 |
| Corn (%) | 33.03 | 20.93 | 31.22 |
| Concentrate supplement (%) | 13.69 | — | — |
| Commercial fermentation concentrate (%) | — | 23.04 | — |
| Vegetable leaf fermentation concentrate (%) | — | — | 22.97 |
| Wheat bran (%) | 2.94 | 4.33 | 1.49 |
| Soybean meal (%) | 13.17 | 14.85 | — |
| Stone powder (%) | 0.17 | 0.08 | — |
| Premix[1)] (%) | 2.53 | 0.17 | 0.43 |
| NaCl (%) | 0.76 | — | 0.46 |
| Total (%) | 100.00 | 100.00 | 100.00 |
| Nutrient levels | | | |
| DE（MJ/kg） | 10.62 | 10.86 | 10.83 |
| CP (%) | 15.38 | 15.20 | 15.91 |
| NDF (%) | 2.87 | 17.96 | 17.08 |
| ADF (%) | 7.79 | 9.53 | 10.18 |
| Ca (%) | 0.60 | 0.46 | 0.62 |
| TP(%) | 0.37 | 0.39 | 0.49 |
